# Identify Function of WASL in Prognosis of Cervical Cancer Based on Omics Data

**DOI:** 10.3389/fcell.2021.670890

**Published:** 2021-06-08

**Authors:** Jinxuan Hou, Chen Chen, Yingying Hu, Qing Gong, Lijuan Gan, Yu Xu

**Affiliations:** ^1^Department of Thyroid and Breast Surgery, Zhongnan Hospital of Wuhan University, Wuhan, China; ^2^Human Genetics Resource Preservation Center of Wuhan University, Wuhan, China; ^3^Human Genetics Resource Preservation Center of Hubei Province, Wuhan, China; ^4^Department of Obstetrics and Gynecology, The Fourth Affiliated Hospital, Zhejiang University School of Medicine, Yiwu, China; ^5^Department of Obstetrics and Gynecology, Zhongnan Hospital of Wuhan University, Wuhan, China; ^6^Department of Gynecological Oncology, Zhongnan Hospital of Wuhan University, Hubei Key Laboratory of Tumor Biological Behaviors, Hubei Cancer Clinical Study Center, Wuhan, China; ^7^Department of Radiation and Medical Oncology, Zhongnan Hospital of Wuhan University, Wuhan, China

**Keywords:** WASL, cervical cancer, prognosis, microarray, omics

## Abstract

**Objective:**

To clarify the clinical relevance of WASP like actin nucleation promoting factor (WASL) in patients with cervical cancer and associated mechanisms.

**Methods and Materials:**

We obtained high prediction accuracy and determined the correlation between the expression of WASL and the clinical characteristics of cervical cancer patients. Differentially expressed genes (DEGs) were identified using microarray. Gene ontology (GO) enrichment analysis and gene set enrichment analysis (GSEA) were performed to determine potentially relevant mechanisms related to the prognostication ability of WASL expression.

**Results:**

Chi-square test and multivariable logistic regression analysis suggested that lower expression of WASL was associated with lower pathological stage (chi-square test: *p* = 0.022, chi-square = 9.613; logistic regression: OR = 0.869, 95% CI: 0.756–0.991, *p* = 0.041). Patients in the WASL high expression group have worse overall survival (OS) [hazard ratio (HR): 0.555, 95% CI: 0.348–0.884, log-rank *p* = 0.012] and recurrence-free survival (RFS) (HR = 0.449, 95% CI: 0.215–0.934, log-rank *p* = 0.028) compared with those in the WASL low expression group. Univariate and multivariable Cox proportional hazards regression model suggested that WASL expression was an independent prognostic factor for predicting OS and RFS in cervical cancer. DEGs were mostly enriched GO terms related to DNA replication or the proliferation of tumor cells. The results of GSEA suggested samples in the WASL knockdown group were enriched in glycolysis, TNF-α signaling via NFkB, mTORC1 signaling, and Wnt/β-catenin signaling.

**Conclusions:**

WASL expression was associated with the pathological stage, and it might be an independent prognostication factor in patients with cervical cancer. Knockdown of WASL might be correlated with biological processes such as glycolysis, TNFα signaling, mTOR signaling, and Wnt/β-catenin signaling.

## Introduction

Cervical cancer represents one of the most frequent malignancies in the female reproductive system (Small et al., 2017). Cervical cancer has the highest incidence in developing countries and represents the leading cause of cancer-related deaths in women in those countries (Pedersen et al., 2018). Meanwhile, there are approximately 500,000 new cases and 280,000 deaths of cervical cancer worldwide annually. Treatments of cervical cancer are tailored by stage (Mboumba Bouassa et al., 2017; Pedersen et al., 2018). For patients with early cervical cancer (carcinoma *in situ*, stages 1 and 2 cervical cancer), surgery to remove the tumor, the cervix, and some or all of the womb (conization, hysterectomy with or without bilateral salpingo-oophorectomy, radical trachelectomy) or radiotherapy (internal radiation therapy, radiation therapy with or without chemotherapy) or a combination of both is often recommended. For patients with advanced cervical cancer, radiation therapy as palliative therapy to relieve symptoms with or without chemotherapy, targeted therapy, and surgery to remove pelvic lymph nodes are often recommended (Li et al., 2016; Hu and Ma, 2018). Despite continuous improvements in surgical techniques, radiotherapy equipment, etc., the therapeutic effect on patients with cervical cancer has not improved fundamentally in the past 40 years. The overall 5-year survival rate of cervical cancer patients is about 40%, and it sharply decreases to about 16.5% for patients with advanced or metastatic cervical cancer (Mapanga et al., 2018; Alimena et al., 2019).

With the advancement of technology, many high-throughput sequencing data and phenotypic data based on cervical cancer have been published, and new algorithms have emerged (Liu et al., 2018; Yu et al., 2021), which has provided new possibilities for developing new molecular markers and personalized treatments for cervical cancer.

WASL, also known as WASP, like actin nucleation promoting factor, is a member of the Wiskott–Aldrich syndrome (WAS) protein family. Our previous study suggested that overexpression of WASL served as an oncogene in cervical cancer by promoting the invasion and migration of cervical cancer cells *in vitro* (Hou et al., 2017). However, the effect and associated mechanisms of WASL on the prognosis of patients with cervical cancer remains unclear.

Therefore, in the present study, we identified the prognostication role and potential mechanism of WASL in clinical settings.

## Materials and Methods

### Cervical Cancer Gene Expression Studies

The Cancer Genome Atlas (TCGA) cervical cancer (TCGA-CESC), measured using the Illumina HiSeq 2000 RNA Sequencing platform by the University of North Carolina TCGA genome characterization center (Cancer Genome Atlas Research Network [CGARN], Albert Einstein College of Medicine [AECM], Analytical Biological Services [ABS], Barretos Cancer Hospital [BCH], Baylor College of Medicine [BCM], Beckman Research Institute of City of Hope [BRICH], et al., 2017), contained 290 cervical cancer samples. The level 3 data of TCGA-CESC and the associated clinical information were downloaded from University of California, Santa Cruz (UCSC) Xena^1^. The expression values of the cervical cancer samples were in log_2_ (*x* + 1) transformed RNA-Seq by expectation-maximization (RSEM) normalized count. A total of 290 patients with cervical cancer in TCGA-CESC were included in this study, of which 149 patients were younger than or were 50 years old, and 141 patients were older than 50 years. There were 159 patients with stage 1, 64 patients with stage 2, 41 patients with stage 3, and 20 patients with stage 4 cancer. There are 18 people in Grade 1, 129 in Grade 2, and 117 in Grade 3.

### Analyzing the Relationship Between the Expression of WASL and the Clinical Outcomes of Cervical Cancer Patients

Cervical patients in the TCGA-CESC cohorts were divided into WASL low expression group and WASL high expression group based on the optimal cutoff (see below). Then, chi-square test and logistic regression were performed to analyze the relationships between the expression of WASL and the age, pathological stage, grade, Eastern Cooperative Oncology Group (ECOG) score, and number of childbirths of cervical patients. Time-dependent receiver operating characteristic curve (ROC) (Kamarudin et al., 2017) was performed to identify the optimal cutoff point that divided cervical cancer patients into WASL low expression group and WASL high expression group. To clarify the prognostic role of WASL, Kaplan–Meier and univariate and multivariable Cox proportional hazards regression model were conducted on the overall survival (OS) and recurrence-free survival (RFS) of cervical cancer patients.

### Lentiviral Vector Transfection

The recombinant lentivirus gene transfer vector targeting WASL (LV-WASL-RNAi) and the lentiviral vector LV-CON049-NC were synthesized by Shanghai Genechem Co. Ltd. The LV-WASL-RNAi and LV-CON049-NC titers were 5 × 10^8^ and 2 × 10^8^ infectious U/ml, respectively. Hela cells were infected with viral supernatant and considered to be knockdown group and normal control group. Fluorescence microscopy was used to detect the fluorescence signal. Each experiment was conducted three times. According to the manufacturer’s protocol, qRT-PCR was conducted using iQTM SYBR^®^ Green PCR supermix (1708880, Bio Red) to determine the transfection efficiency. The primers used were as follows: GAPDH: TGACTTCAACAGCGACACCCA (forward), CACCCTGTTGCTGTAGCCAA (reverse); WASL: GAACGAGTCCCTCTTCACTTTC (forward), GTTCCGATC TGCTGCATATAACT (reverse). As shown in Supplementary Figure 1, WASL gene knockdown efficiency reached 81.3% in the knockdown group (1 ± 0.033 vs 0.187 ± 0.020, *p* < 0.001)

### Immunohistochemistry in Tissue Microarray

A tissue microarray of cervical cancer samples was acquired from the Shanghai Outdo Biotech Company (Shanghai China). The microarray is composed of 126 cervical cancer tissues and 42 paracancerous tissues. First, antigen retrieval was performed in citrate buffer (pH 6.0) and treated with 3% H_2_O_2_ after deparaffinization and rehydration. Then, the slides were incubated with primary antibody (ab126626; Abcam) and secondary antibody continuously at 4°C overnight until visualization with peroxidase and 3,3′-diaminobenzidine tetrahydrochloride. After that, the expression of WASL in the cervical cancer tissues from the tissue microarray could be blindly quantified. Finally, the average integrated optical density (IOD) per stained area (μm^2^) (IOD/area) for positive staining could be calculated by the Image-pro Plus software 6.0.

### Gene Expression Profiling

The total RNA of three pairs of cervical cancer Hela cells was extracted and converted to cDNAs, which were used to synthesize double-stranded DNA (dsDNA) templates. For RNA extraction, Trizol method was used to extract total RNA from the samples. The total RNA extracted was subjected to NanoDrop 2000 (1.7 < A260/A280 < 2.2) and Agilent Bioanalyzer 2100 (RIN ≥ 7.0 and 28S/18S > 0.7) for quality control, and qualified samples entered the chip experiment. Then, the dsDNA was used to synthesize biotin-labeled cRNA using GeneChip 3’IVT Express Kit (Affymetrix). Finally, the biotin-labeled cRNA was hybridized to Affymetrix Human Gene Expression Array (PrimeView) according to the manufacturer’s protocol. The probe cell intensity was normalized using robust multi-array average (RMA) methods in the R/bioconductor package “affy” (Gautier et al., 2004). R package “limma” (Ritchie et al., 2015) was used to identify differentially expressed genes (DEGs) between the knockdown group and control group. Absolute fold change >1.3 and *p* < 0.05 were treated as the criteria for the screening of DEGs.

### Functional Analysis

To make clear the function of the DEGs, gene ontology (GO) enrichment analysis was performed using the R/bioconductor package “clusterProfiler” (Yu et al., 2012). GO terms meeting *p* and false discovery rate (FDR) less than 0.05 were considered to be significant. Gene Set Enrichment Analysis (GSEA) (Subramanian et al., 2005), a systems biology approach, could be used to determine whether a defined gene set shows statistically significant, concordant differences between two or more biological groups. Thus, we conducted GSEA (Mootha et al., 2003; Subramanian et al., 2005) on the knockdown group and control group based on the expression profile of the three pairs of cervical cancer cells. Hallmark gene sets v.7.0 was used as a reference gene set. Other parameters were set as default. Gene sets meeting *p* < 0.05 and FDR < 0.25 were considered to be significantly enriched.

## Results

### Relationships Between WASL Expression and the Clinical Characteristics of Patients With Cervical Cancer

Based on the optimal cutoff point (10.4851), the cervical cancer samples in the TCGA-CESC study were categorized into WASL high expression group (n = 114) and WASL low expression group (*n* = 176). As shown in Table 1, the chi-square test and multivariable logistic regression analysis suggested that lower expression of WASL was associated with a lower pathological stage (chi-square test: *p* = 0.022, chi-square = 9.613; logistic regression: OR = 0.869, 95% CI: 0.756–0.991, *p* = 0.041), while the expression of WASL was not correlated with other clinical characteristics (age, grade, ECOG score, and number of childbirths).

**TABLE 1:** The associations between the expression of WAS expression and clinical characteristic of patients with cervical cancer in TCGA-CESC.

	WASL expression		Chi-square test		Logistic regression			
	High	Low	*p*-Value	Chi-square	OR	LCI	UCI	*p*-Value
**Age (years)**								
≤50	63	86	0.345	0.893	1.012	0.984	1.04	0.41
>50	51	90						
**Stage**								
1	75	84	0.022	9.613	0.869	0.756	0.991	0.041
2	17	47						
3	13	28						
4	7	13						
**Grade**								
G1	9	9	0.08	5.049	0.621	0.349	1.086	0.098
G2	57	72						
G3	37	80						
**ECOG**								
0	45	67	0.541	1.228	1.374	0.831	2.294	0.214
1	29	49						
2∼4	6	5						
**Number of childbirths**								
≤3	72	109	1	0	1.002	0.838	1.191	0.985
>3	29	43						

### Protein Level of WASL Was Significantly Higher in Cervical Cancer Compared With That in Their Adjacent Normal Tissues

**FIGURE 1:**
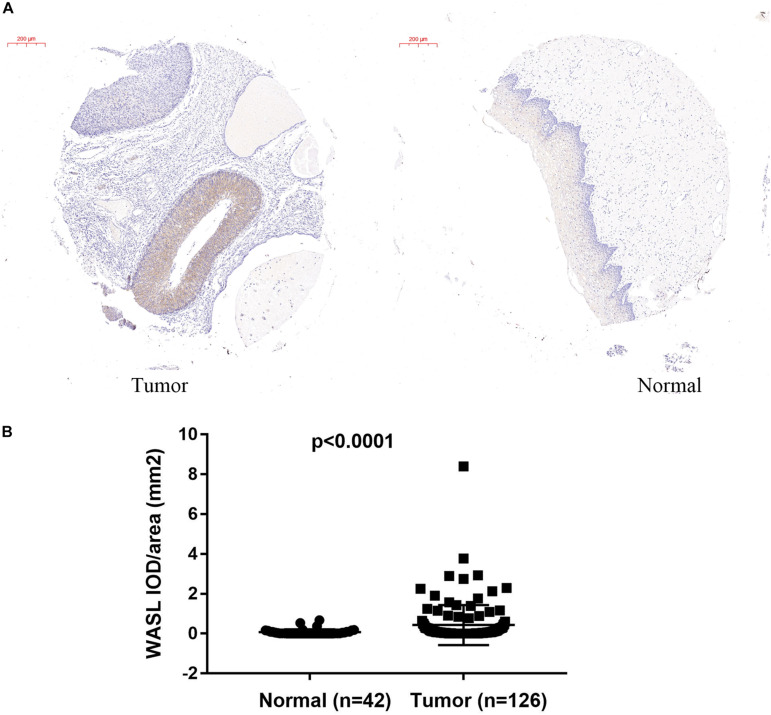
Immunohistochemistry (IHC) staining in tissue microarray showing increased WASL protein level in cervical cancer tissues. The representative images of IHC are shown in **(A)**, while the quantification of WASL protein level is summarized in **(B)**. Scale bar in **(A)**, 200 μm. Student’s *t*-test was performed and data represent means ± SEM in **(B)**. *P* < 0.0001.

To identify the upregulation of WASL in cervical cancer, we detected the protein level of WASL in tissue microarray by immunohistochemistry (IHC) using WASL antibodies. The average IOD per stained area (μm^2^) (IOD/area) was used to calculate a positive signal. As expected, the protein level of WASL was significantly higher in cervical cancer tissues compared with that in their adjacent normal tissues (Figures 1A,B).

### Lower Expression of WASL Was Associated With Better Survival of Cervical Patients

**FIGURE 2:**
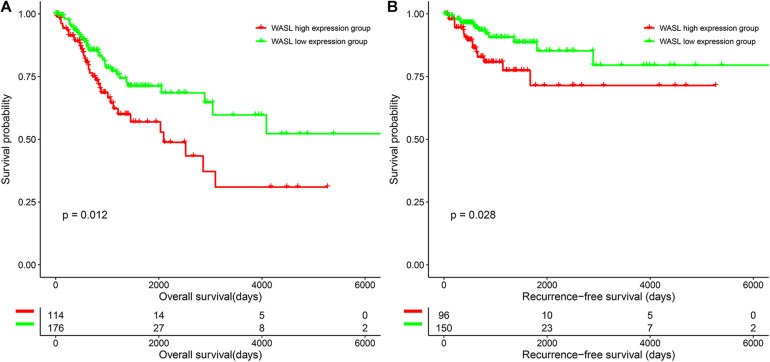
Survival differences of patients in the WASL low expression group and WASL high expression group. **(A)** Overall survival. **(B)** Recurrence-free survival.

To characterize the prognostic role of WASL, we performed survival analysis of cervical cancer patients based on the expression of WASL. As shown in Figure 2, patients in the WASL high expression group had worse OS (HR: 0.555, 95% CI: 0.348–0.884, log-rank *p* = 0.012, Figure 2A and Table 2) and RFS (HR = 0.449, 95% CI: 0.215–0.934, log-rank *p* = 0.028, Figure 2B and Table 3) compared with those in the WASL low expression group. In addition, the univariate and multivariable Cox proportional hazards regression model suggested that WASL expression was an independent prognostic factor for predicting OS (Table 2) and RFS (Table 3) in cervical cancer.

**TABLE 2:** Univariate and multivariable Cox proportional hazards regression on the overall survival in TCGA-CESC.

Characteristic	Univariate analysis				Multivariable analysis			
	HR	LCI	UCI	*p*-Value	HR	LCI	UCI	*p*-Value
WASL	0.555	0.348	0.884	0.013	0.23	0.103	0.514	<0.001
Age	1.017	1	1.035	0.057	1.019	0.986	1.053	0.261
Stage	1.141	1.059	1.23	0.001	1.261	1.098	1.447	0.001
Grade	1.009	0.652	1.56	0.969	1.212	0.639	2.3	0.557
ECOG	1.769	1.165	2.688	0.007	1.722	1.067	2.779	0.026
Number of childbirths	1.042	0.935	1.162	0.458	1.013	0.845	1.214	0.891

**TABLE 3:** Univariate and multivariable Cox proportional hazards regression on the recurrence-free survival in TCGA-CESC.

Characteristics	Univariate analysis				Multivariable analysis			
	HR	LCI	UCI	*p*-Value	HR	LCI	UCI	*p*-Value
WASL	0.449	0.215	0.934	0.032	0.337	0.119	0.956	0.041
Age	1.005	0.977	1.033	0.735	1.026	0.98	1.073	0.273
Stage	0.98	0.859	1.118	0.767	0.986	0.795	1.223	0.898
Grade	1.361	0.711	2.606	0.352	2.685	1.033	6.976	0.043
ECOG	2.383	1.461	3.887	0.001	2.116	1.191	3.76	0.011
Number of childbirths	0.941	0.771	1.148	0.547	0.939	0.713	1.237	0.656

### Differentially Expressed Genes Between WASL Low Expression Group and WASL High Expression Group

**FIGURE 3:**
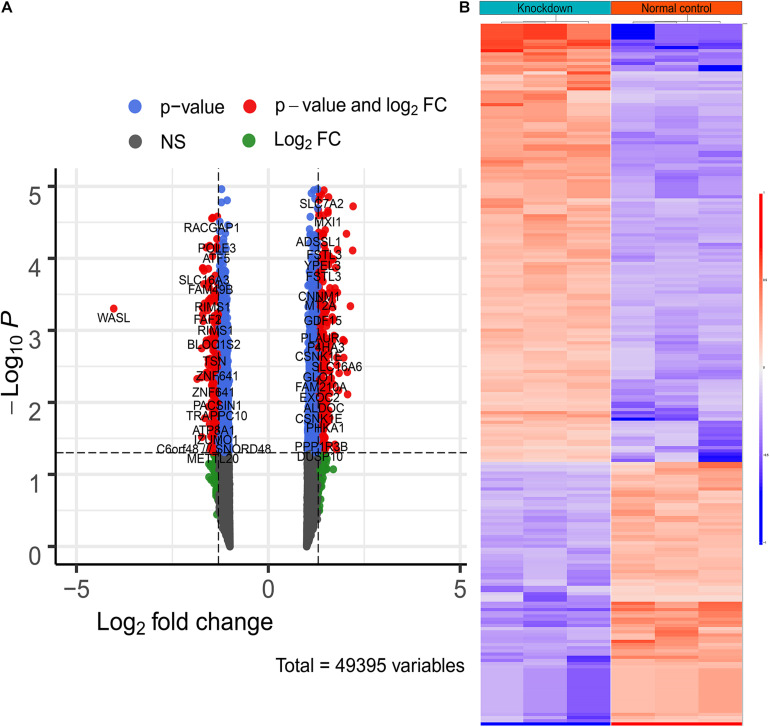
The differentially expressed genes between WASL knockdown group and normal control group. **(A)** Volcano plot depicting the overall distribution of gene expressions between the two groups. **(B)** heatmap depicting the expression levels of differentially expressed genes.

As mentioned above, the raw expression signals of the expression profile were normalized using RMA methods. Then, the DEGs between the two groups were calculated via “limma.” Consequently, a total of 221 DEGs (including 83 down-regulated genes and 138 up-regulated genes) were identified according to the inclusion criteria (Figure 3 and Supplementary Table 1).

### Functional Enrichment Analysis

**FIGURE 4:**
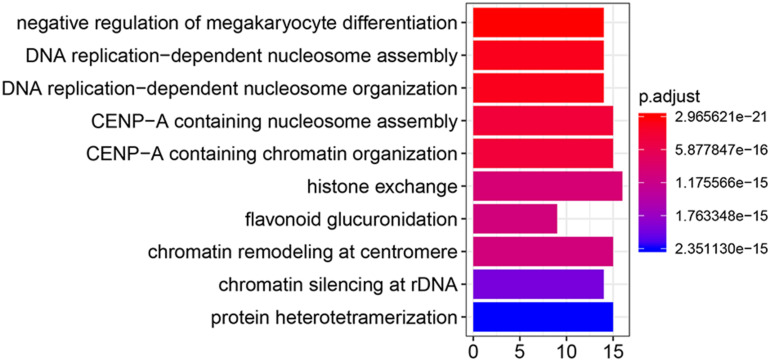
Gene ontology enrichment analysis of differentially expressed genes.

**FIGURE 5:**
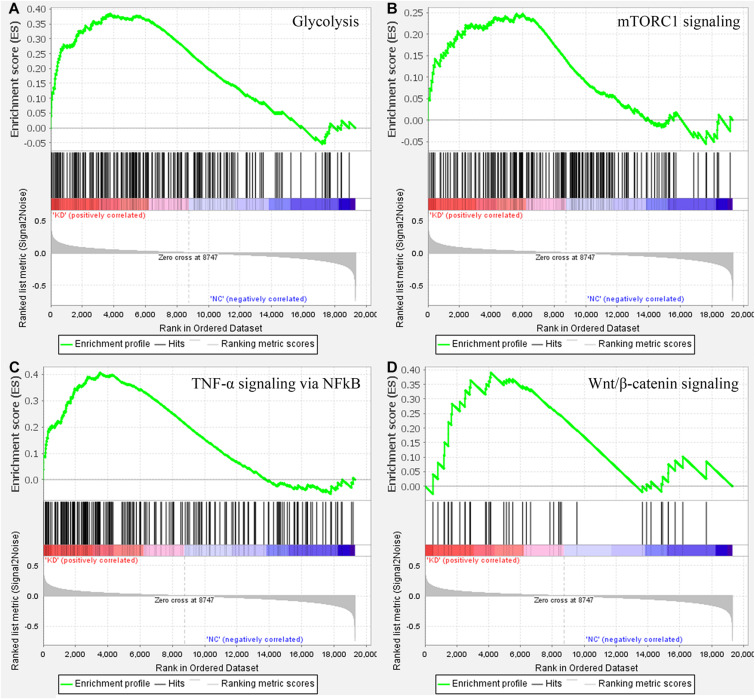
Gene set enrichment analysis on the cervical samples with WASL knockdown. **(A)** glycolysis, **(B)** TNF-a signaling via NF-kB, **(C)** mTORC1 signaling, **(D)** Wnt/b-catenin signaling.

To make clear the biological meaning of the DEGs, GO enrichment analysis was performed on the DEGs. As shown in Figure 4, the DEGs were mostly enriched in “negative regulation of megakaryocyte differentiation,” “DNA replication-dependent nucleosome assembly,” “DNA replication-dependent nucleosome organization,” “CENP-A containing nucleosome assembly,” “CENP-A containing chromatin organization,” “histone exchange,” “flavonoid glucuronidation,” “chromatin remodeling at centromere,” “chromatin silencing at rDNA,” and “protein heterotetramerization.” Thus, the results of GO enrichment analysis suggested that DEGs were related with DNA replication or the proliferation of tumor cells. In addition, the results of GSEA suggested samples in the WASL knockdown group were enriched in glycolysis (normalized enrichment score (NES) = 1.429, *p* < 0.0001, FDR = 17.754%, Figure 5A), TNF-α signaling via NF-κB (NES = 1.475, *p* < 0.0001, FDR = 19.236%, Figure 5B), mTORC1 signaling (NES = 1.489, *p* < 0.0001, FDR = 23.682%, Figure 5C), and Wnt/β-catenin signaling (NES = 1.449, *p* < 0.0001, FDR = 18.865%, Figure 5D).

## Discussion

In the present study, we demonstrated that the expression of WASL was significantly correlated with the pathological stage of cervical cancer patients, and lower expression of WASL was associated with better survival of cervical cancer patients. Actually, WASL was reported to be associated with the biological behaviors of several human cancers. Schwickert et al. suggested that the knockdown of WASL significantly decreased the invasiveness of human breast cancer cells (Schwickert et al., 2015). Peng et al. suggested down-regulation of WASL suppressed the migration of melanoma cells (Peng et al., 2019). Li et al. indicated that MicroRNA-214-5p prohibited the invasion and migration of liver cancer cells through its down-regulation of WASL (Li H. et al., 2018). These studies suggested that WASL might play a role of promoting the progression of cervical cancer cells.

Gene ontology enrichment analysis based on biological process suggested that the DEGs between WASL knockdown group and normal control suggested that the DEGs were mostly enriched in DNA replication or the proliferation of tumor cells, which might explain to some extent why WASL could be used as a prognostic marker for patients with cervical cancer. The results of GSEA suggested that samples in the WASL knockdown group were mostly enriched in glycolysis (genes encoding proteins involved in glycolysis and gluconeogenesis), TNF-α signaling via NF-êB (genes regulated by NF-êB in response to TNF), mTORC1 signaling (genes up-regulated through activation of mTORC1 complex), and Wnt/β-catenin signaling (genes up-regulated by activation of WNT signaling through accumulation of β-catenin). Glycolysis is an important way for organisms (including tumor cells) to obtain energy (Cheng et al., 2013). As a proinflammatory cytokine, TNFα had been reported to be associated with cell proliferation, differentiation, apoptosis, and immune response. Moreover, Li et al. indicated that TNF-α induced the apoptosis of cervical cells (Li J. et al., 2018). Rashmi et al. suggested that AKT inhibitors suppress the proliferation of cervical cancer cells via disruption of mTOR signaling and glucose uptake (Rashmi et al., 2014). Meanwhile, Hsu et al. (2019) reported that activation of Wnt/β-catenin signaling promoted the growth of cervical cancer. Thus, it indicated the knockdown of WASL might be correlated with biological processes such as glycolysis, TNFα signaling, mTOR signaling, and Wnt/β-catenin signaling, which, in return, might suppress the progress of cervical cancer in clinical settings.

In summary, the expression of WASL was associated with the pathological stage, OS, and RFS, and it might be an independent prognostication factor in patients with cervical cancer. DEGs between WSAL knockdown group and normal controls were associated with DNA replication- and cell proliferation-related GO terms. Knockdown of WASL might be correlated with biological process such as glycolysis, TNFα signaling, mTOR signaling, and Wnt/β-catenin signaling.

## Data Availability Statement

The data presented in the study are deposited in the Github repository, which could be accessed via https://github.com/xuyulab/cervical-cancer.

## Author Contributions

YX: conception. JH, CC, and YH: interpretation or analysis of data. JH and CC: preparation of the manuscript. JH, QG, LG, and YH: revision for important intellectual content. YX: supervision. All authors contributed to the article and approved the submitted version.

## Conflict of Interest

The authors declare that the research was conducted in the absence of any commercial or financial relationships that could be construed as a potential conflict of interest.

## Footnotes

1 https://xenabrowser.net/datapages/

## Supplementary Material

Supplementary Figure 1 RT-PCR depicting the knockdown efficiency of WASL.

Supplementary Table 1 Differentially expressed genes between WASL knockdown group and normal control group.
