# Supplementary figures and images for: Identify Function of WASL in Prognosis of Cervical Cancer Based on Omics Data
Source: Front Cell Dev Biol. 2021 Jun 8;9:670890. doi: 10.3389/fcell.2021.670890 (PMC8248809; doi:10.3389/fcell.2021.670890)

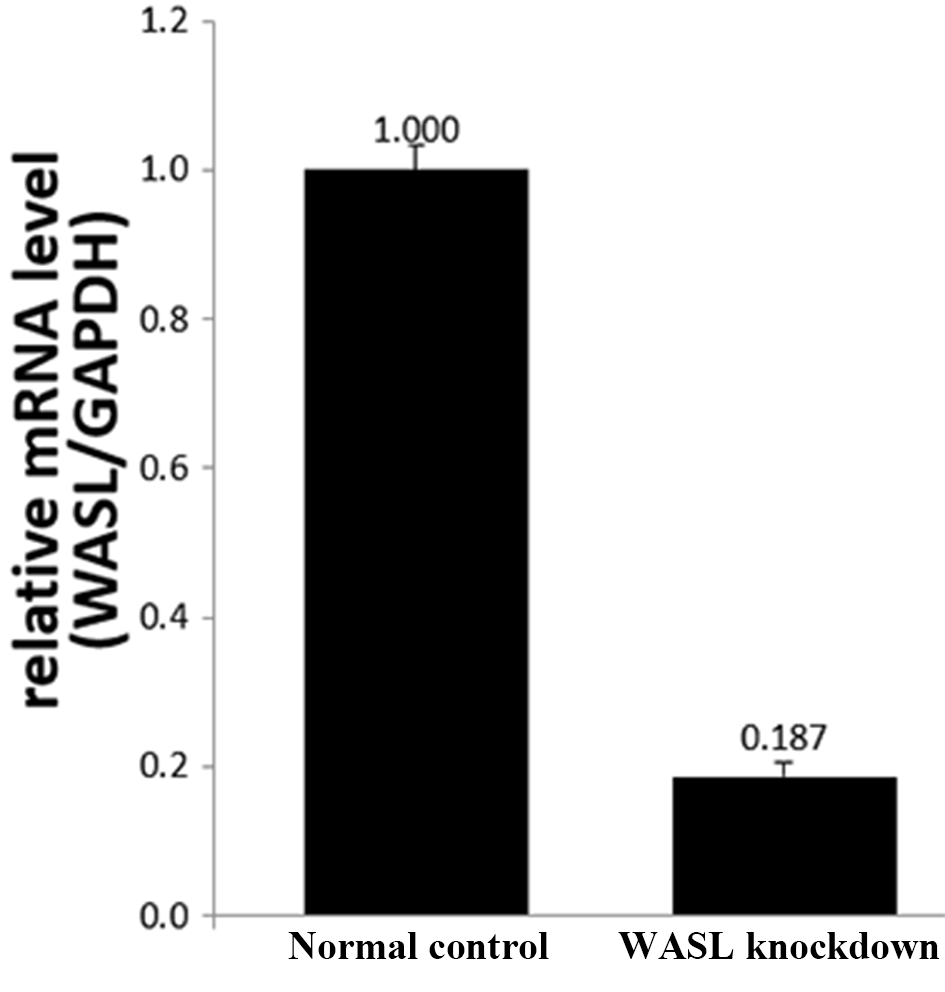

Supplement: Supplementary Figure 1 — RT-PCR depicting the knockdown efficiency of WASL. [file Image_1.TIF]
