# Supplementary material for: Identify Function of WASL in Prognosis of Cervical Cancer Based on Omics Data
Source: Front Cell Dev Biol. 2021 Jun 8;9:670890. doi: 10.3389/fcell.2021.670890 (PMC8248809; doi:10.3389/fcell.2021.670890)
Supplement: Supplementary Table 1 — Differentially expressed genes between WASL knockdown group and normal control group. [file Table_1.docx]

**Supplementary Table 1** Differentially expressed genes between WASL knockdown group and normal control group

| Gene | Corrected p-value | Fold change | Log Fold change |
| --- | --- | --- | --- |
| PROCR | 0.000989 | 1.33849 | 0.420606 |
| SLC16A3 | 0.000989 | -1.7017 | -0.76698 |
| FBXO21 | 0.000989 | 1.57075 | 0.651454 |
| SLC7A2 | 0.000989 | 1.388477 | 0.473503 |
| OLR1 | 0.000989 | -1.93923 | -0.95548 |
| MXI1 | 0.001035 | 1.554989 | 0.636905 |
| BNIP3 | 0.001035 | 1.303368 | 0.382244 |
| SERPINB2 | 0.001035 | 2.209383 | 1.143644 |
| FAM98A | 0.001035 | -1.45191 | -0.53795 |
| FTH1 | 0.001035 | 1.441866 | 0.527937 |
| SCAPER | 0.001205 | 1.482239 | 0.567778 |
| MBOAT1 | 0.001205 | 1.378428 | 0.463024 |
| ADSSL1 | 0.001205 | 1.312155 | 0.391938 |
| CA9 | 0.001205 | 2.036447 | 1.026055 |
| GPRC5A | 0.001205 | 1.419873 | 0.505762 |
| POLE3 | 0.001257 | -1.33978 | -0.422 |
| FSTL3 | 0.00132 | 1.456699 | 0.542703 |
| STC1 | 0.00132 | 2.197536 | 1.135887 |
| MRPL20 | 0.00132 | -1.34389 | -0.42642 |
| ARNT2 | 0.00132 | 1.803163 | 0.85053 |
| FAM49B | 0.00132 | -1.53231 | -0.61571 |
| CCL5 | 0.00132 | -1.36392 | -0.44776 |
| ATF5 | 0.00132 | -1.3543 | -0.43755 |
| HEY1 | 0.00132 | 1.486314 | 0.571739 |
| SERPINB4 | 0.001388 | 1.586471 | 0.665822 |
| SERPINB3 | 0.001388 | 1.586471 | 0.665822 |
| YPEL3 | 0.001388 | 1.414123 | 0.499907 |
| ANKRD37 | 0.001388 | 1.487085 | 0.572487 |
| C1R | 0.001388 | 1.42593 | 0.511903 |
| LOX | 0.001388 | 1.57299 | 0.65351 |
| CPD | 0.001388 | 1.380717 | 0.465418 |
| CDKN1C | 0.001388 | 1.317013 | 0.397269 |
| HPSE | 0.001388 | 1.343396 | 0.425885 |
| NNT | 0.001399 | 1.568872 | 0.649728 |
| ELL2 | 0.001399 | 1.305896 | 0.38504 |
| ENTPD7 | 0.001399 | 1.306699 | 0.385927 |
| RAP1A | 0.001456 | -1.56306 | -0.64438 |
| UBE2Z | 0.001456 | -1.71359 | -0.77702 |
| TIPARP | 0.001459 | 1.318162 | 0.398528 |
| WNT5A | 0.001529 | -1.38419 | -0.46904 |
| CDCA7L | 0.001567 | 1.367844 | 0.451904 |
| SPTBN1 | 0.001633 | 1.330188 | 0.41163 |
| RAB2A | 0.00165 | -1.33329 | -0.41499 |
| RACGAP1 | 0.001688 | -1.67741 | -0.74623 |
| PCID2 | 0.001755 | 1.603856 | 0.681545 |
| SLC2A14 | 0.001782 | 1.579406 | 0.659382 |
| LEPROTL1 | 0.001782 | -1.3367 | -0.41868 |
| IFT74 | 0.001782 | -1.38967 | -0.47474 |
| LOC100509484 | 0.001782 | 1.424133 | 0.510084 |
| PLIN2 | 0.001782 | 1.424133 | 0.510084 |
| GLA | 0.001782 | -1.37103 | -0.45526 |
| EIF4EBP2 | 0.001782 | -1.41628 | -0.50211 |
| SLC2A3 | 0.001782 | 1.579406 | 0.659382 |
| GPR56 | 0.001782 | 1.303036 | 0.381877 |
| GLO1 | 0.001782 | 1.342942 | 0.425397 |
| THRA | 0.00182 | -1.37021 | -0.4544 |
| C8orf4 | 0.001844 | 1.680351 | 0.748763 |
| MT2A | 0.001881 | 1.358955 | 0.442497 |
| UBA3 | 0.001898 | -1.38598 | -0.47091 |
| HLA-A | 0.001898 | 1.340969 | 0.423275 |
| CPA4 | 0.001898 | -1.35806 | -0.44155 |
| CDK6 | 0.001898 | -1.31904 | -0.39949 |
| ATP6V1A | 0.001898 | -1.50423 | -0.58902 |
| NUP98 | 0.001898 | -1.43392 | -0.51997 |
| SLC39A8 | 0.001898 | -1.39583 | -0.48112 |
| ISG20 | 0.001974 | 1.357589 | 0.441047 |
| CADM1 | 0.001979 | 1.366696 | 0.450692 |
| AP2B1 | 0.001979 | -1.66185 | -0.73279 |
| VWA5A | 0.001979 | 1.324972 | 0.405961 |
| RHOU | 0.001983 | 1.506525 | 0.591224 |
| UGT1A7 | 0.001989 | 2.138839 | 1.096828 |
| UGT1A9 | 0.001989 | 2.138839 | 1.096828 |
| UGT1A8 | 0.001989 | 2.138839 | 1.096828 |
| UGT1A3 | 0.001989 | 2.138839 | 1.096828 |
| UGT1A5 | 0.001989 | 2.138839 | 1.096828 |
| SHMT2 | 0.002 | 1.318575 | 0.398979 |
| ZNF331 | 0.002 | 1.356813 | 0.440222 |
| RAB8B | 0.002 | -1.71558 | -0.7787 |
| ADM | 0.002021 | 1.324227 | 0.40515 |
| CRISPLD2 | 0.002021 | 1.364591 | 0.448468 |
| WASL | 0.002021 | -4.02926 | -2.01052 |
| LOC100653157 | 0.002027 | 1.410056 | 0.495752 |
| TGFBI | 0.002027 | 1.410056 | 0.495752 |
| LOC100652886 | 0.002027 | 1.410056 | 0.495752 |
| FAM13A | 0.002027 | 1.353757 | 0.436969 |
| ANGPTL4 | 0.002064 | 1.51142 | 0.595905 |
| SSBP2 | 0.002064 | 1.50441 | 0.589198 |
| FAF2 | 0.002064 | -1.58253 | -0.66223 |
| GDF15 | 0.002073 | 1.420395 | 0.506292 |
| ZMIZ1 | 0.002116 | 1.487674 | 0.573059 |
| EMR2 | 0.002149 | 1.365868 | 0.449818 |
| FOLR1 | 0.002175 | 1.424193 | 0.510145 |
| PTGES | 0.002177 | -1.30425 | -0.38322 |
| RHOB | 0.002183 | 1.326895 | 0.408055 |
| ARRDC3 | 0.002197 | 1.386938 | 0.471904 |
| ZNF652 | 0.002197 | -1.40701 | -0.49263 |
| OASL | 0.002244 | -1.31545 | -0.39556 |
| DNAJC14 | 0.002244 | -1.30048 | -0.37905 |
| ZNF673 | 0.002261 | 1.398915 | 0.484308 |
| GTF2H1 | 0.002261 | -1.30161 | -0.38029 |
| TMPRSS3 | 0.002261 | 1.334238 | 0.416016 |
| CLK1 | 0.002268 | 1.310285 | 0.389881 |
| NDRG1 | 0.002268 | 1.687664 | 0.755028 |
| UGT1A10 | 0.002285 | 1.450499 | 0.536549 |
| UGT1A1 | 0.002285 | 1.450499 | 0.536549 |
| UGT1A6 | 0.002285 | 1.450499 | 0.536549 |
| UGT1A4 | 0.002285 | 1.450499 | 0.536549 |
| RIMS1 | 0.002294 | -1.3741 | -0.45849 |
| ERRFI1 | 0.002298 | 1.499076 | 0.584073 |
| RAB31 | 0.002409 | 1.324933 | 0.405919 |
| TMEM45A | 0.002481 | 1.301403 | 0.380068 |
| IGFBP3 | 0.002492 | 1.517093 | 0.60131 |
| DUSP5 | 0.002503 | 1.349371 | 0.432287 |
| SYDE1 | 0.002596 | 1.335854 | 0.417762 |
| PLAUR | 0.002616 | 1.348292 | 0.431133 |
| PI16 | 0.00269 | -1.365 | -0.4489 |
| ABI2 | 0.002697 | 1.326501 | 0.407626 |
| ZNHIT1 | 0.002708 | -1.46249 | -0.54842 |
| PODXL | 0.002821 | -1.43462 | -0.52067 |
| PFKFB4 | 0.002856 | 1.425425 | 0.511392 |
| PLEKHG1 | 0.002856 | 1.366402 | 0.450382 |
| BLOC1S2 | 0.002922 | -1.41896 | -0.50484 |
| RAVER2 | 0.003046 | -1.38574 | -0.47066 |
| HK2 | 0.003055 | 1.409467 | 0.49515 |
| TSN | 0.00308 | -1.41248 | -0.49823 |
| CYBRD1 | 0.003095 | -1.32692 | -0.40808 |
| MSMO1 | 0.003157 | 1.305851 | 0.38499 |
| SGTB | 0.003172 | -1.40056 | -0.48601 |
| FAM96A | 0.003177 | -1.61321 | -0.68993 |
| HLA-DMA | 0.003195 | 1.673969 | 0.743273 |
| CD93 | 0.003199 | -1.45625 | -0.54226 |
| EIF4H | 0.003333 | -1.34404 | -0.42657 |
| SIVA1 | 0.003428 | -1.31091 | -0.39057 |
| IDH2 | 0.003428 | 1.325988 | 0.407067 |
| XPR1 | 0.003483 | 1.3366 | 0.418568 |
| KLHDC5 | 0.003504 | -1.45436 | -0.54038 |
| ALDOC | 0.003594 | 1.60063 | 0.67864 |
| B3GALNT2 | 0.003702 | -1.73759 | -0.79709 |
| YY1 | 0.003735 | 1.307323 | 0.386615 |
| CASP4 | 0.003735 | -1.39762 | -0.48298 |
| RNASE4 | 0.00386 | 1.392839 | 0.478028 |
| ATP11C | 0.003964 | -1.37006 | -0.45424 |
| FAM69A | 0.004053 | 1.406413 | 0.492021 |
| SMTN | 0.004083 | 1.571456 | 0.652102 |
| NUPL1 | 0.004087 | 1.467026 | 0.552895 |
| VKORC1L1 | 0.004157 | 1.418743 | 0.504613 |
| KIAA1033 | 0.004157 | -1.31372 | -0.39365 |
| NUCB2 | 0.004157 | 1.390489 | 0.475592 |
| RGS10 | 0.004157 | 1.35811 | 0.4416 |
| CGA | 0.004288 | 1.421713 | 0.50763 |
| SLC16A6 | 0.004451 | 1.786197 | 0.836891 |
| JUN | 0.004503 | 1.394501 | 0.479749 |
| FAM20B | 0.004672 | -1.5629 | -0.64423 |
| MIR3652 | 0.004751 | 1.30593 | 0.385077 |
| HSP90B1 | 0.004751 | 1.30593 | 0.385077 |
| APOC1 | 0.00488 | -1.50043 | -0.58537 |
| FAM46B | 0.005235 | -1.48604 | -0.57148 |
| BIK | 0.005235 | 1.788642 | 0.838864 |
| HIST1H4L | 0.005235 | -1.48004 | -0.56564 |
| HIST1H4K | 0.005235 | -1.48004 | -0.56564 |
| HIST1H4A | 0.005235 | -1.48004 | -0.56564 |
| HIST1H4B | 0.005235 | -1.48004 | -0.56564 |
| HIST1H4E | 0.005235 | -1.48004 | -0.56564 |
| HIST1H4F | 0.005235 | -1.48004 | -0.56564 |
| HIST1H4C | 0.005235 | -1.48004 | -0.56564 |
| HIST1H4D | 0.005235 | -1.48004 | -0.56564 |
| HIST1H4I | 0.005235 | -1.48004 | -0.56564 |
| HIST1H4J | 0.005235 | -1.48004 | -0.56564 |
| HIST1H4H | 0.005235 | -1.48004 | -0.56564 |
| HIST4H4 | 0.005235 | -1.48004 | -0.56564 |
| HIST2H4A | 0.005235 | -1.48004 | -0.56564 |
| HIST2H4B | 0.005235 | -1.48004 | -0.56564 |
| EGLN3 | 0.005446 | 1.369043 | 0.453167 |
| VLDLR | 0.005681 | 1.318872 | 0.399304 |
| PPFIBP1 | 0.005711 | 1.374284 | 0.45868 |
| CSNK1E | 0.005711 | 1.315129 | 0.395204 |
| CCNG2 | 0.005944 | 1.308011 | 0.387375 |
| ZC3H11A | 0.005944 | 1.327282 | 0.408475 |
| KCTD9 | 0.006206 | -1.6099 | -0.68697 |
| PYGL | 0.00628 | 1.844189 | 0.882987 |
| ZHX3 | 0.006432 | 1.319769 | 0.400286 |
| TCL6 | 0.007075 | -1.30861 | -0.38804 |
| APOBEC3B | 0.007137 | 1.37027 | 0.45446 |
| ZNF641 | 0.007949 | -1.42754 | -0.51353 |
| PRMT6 | 0.008053 | 1.306878 | 0.386125 |
| SEPT11 | 0.008533 | 1.490862 | 0.576147 |
| APOLD1 | 0.008983 | 1.306282 | 0.385467 |
| C17orf103 | 0.009065 | 1.409585 | 0.495271 |
| RAB27A | 0.009951 | 1.349344 | 0.432258 |
| RC3H1 | 0.010068 | -1.30745 | -0.38676 |
| SLC25A13 | 0.010292 | -1.41274 | -0.4985 |
| PLSCR4 | 0.010671 | -1.36907 | -0.45319 |
| SLC35G2 | 0.01111 | -1.32721 | -0.4084 |
| WDR26 | 0.011896 | 1.335119 | 0.416968 |
| LONRF1 | 0.011896 | 1.404719 | 0.490282 |
| MCF2L | 0.012357 | 1.387871 | 0.472873 |
| APOBEC3A_B | 0.012503 | 1.382094 | 0.466856 |
| APOBEC3A | 0.012503 | 1.382094 | 0.466856 |
| MAFF | 0.012939 | 1.362145 | 0.445881 |
| OAF | 0.017142 | -1.36234 | -0.44609 |
| CNOT6L | 0.0178 | 1.442966 | 0.529037 |
| LOC100505813 | 0.018663 | -1.32163 | -0.40232 |
| SPARC | 0.018663 | -1.32163 | -0.40232 |
| PDK3 | 0.019217 | 1.601343 | 0.679282 |
| EXOC7 | 0.019217 | 1.314892 | 0.394944 |
| DYX1C1-CCPG1 | 0.023757 | 1.387848 | 0.47285 |
| CCPG1 | 0.023757 | 1.387848 | 0.47285 |
| FAM100B | 0.02378 | 1.327733 | 0.408965 |
| IGFBP7 | 0.024054 | 1.387417 | 0.472402 |
| CEP44 | 0.024984 | 1.331502 | 0.413054 |
| SNX12 | 0.0272 | -1.4145 | -0.50029 |
| DUSP9 | 0.028851 | 1.346824 | 0.429561 |
| DTNA | 0.02896 | 1.31431 | 0.394306 |
| PRDM7 | 0.033349 | 1.374803 | 0.459225 |
| MARCH6 | 0.033349 | -1.30908 | -0.38855 |
| SEPT8 | 0.033749 | 1.452319 | 0.538358 |
| SPRED1 | 0.036969 | 1.449184 | 0.535241 |
| DAAM1 | 0.037223 | 1.415499 | 0.50131 |
| PMS2 | 0.037636 | -1.31484 | -0.39489 |
| PMS2CL | 0.037636 | -1.31484 | -0.39489 |
| CHD2 | 0.037696 | -1.30103 | -0.37965 |
